# Report of the Foreign Relations Committee

**Published:** 1899-03

**Authors:** 


### Domestic Correspondence.

### REPORT OF THE FOREIGN RELATIONS COMMITTEE.

[THE following partial exhibit of the work of the Foreign Relations Committee of the National Association of Dental Faculties will be read with interest. It cannot strictly be classified as a report, but it shows the deep interest this committee has aroused among educators in Illinois and also faintly outlines the energetic work of the chairman, Dr. Barrett.—ED.]

### TO AMERICAN DENTISTS PRACTISING IN EUROPE.

At a special meeting of the Foreign Relations Committee of the National Association of Dental Faculties of America, held in Cincinnati, Ohio, December 27, 28, and 29, 1898, in accordance with the instructions given at the annual meeting of the Association, held in Omaha, Neb., August, 1898, certain of their *confrères* living in Europe were appointed to form the nucleus of an advisory body, the membership of which it is their purpose to increase to the number of three for each of the principal countries of Europe, as soon as they shall become thoroughly convinced as to the best manner of organizing such Board, and fully informed concerning nominations for membership therein.

The Foreign Relations Committee feels itself restricted in its action by the instructions given it by the National Association, and cannot at present clearly see its way to do more than to lay the foundation for future more comprehensive action. It believes that any precipitancy on its part, in the absence of a full and clear comprehension of the exact status of American dentistry in Europe, might do great injury to the cause of American dental education, and prejudice us greatly in the eyes of foreign professional men. The members also realize that until there is a better understanding of professional affairs in Europe by Americans in this country, it would be easy to injure our colleges by creating a prejudice that would be baseless and unjust. No possible harm can result from the exercise of great care, and even from delay, on our part in the completion of the appointment to this Board, while radical action in the absence of definite knowledge would be certain to work evil.

Hence the Committee has not felt itself justified in doing more at present than to make a few appointments that are entirely unopposed, and to go no further than to commit to such members of the Foreign Board the responsibility of examining the credentials of students making application from foreign countries for matriculation in American dental colleges, the advising of the Foreign Relations Committee of the requirements demanded for practice in such countries, the number, names, and professional status of the holders of American dental degrees abroad, and the giving of such other information as may prove of benefit to the National Association of Dental Faculties. For the information of such Foreign Board the Committee has unanimously adopted the following expression of opinion:

*First.* The proposed Board shall be known as the European Advisory Board of the Foreign Relations Committee of the National Association of Dental Faculties of America.

*Second.* Its objects shall be to ascertain the standing and reputation of institutions in foreign countries giving instruction in dental subjects, the character of instruction imparted, the different courses of study, the length of term, the requirements for admission, and the form of certificate given entitling the holder to practise dentistry in such foreign countries.

*Third.* To examine the certificates of Europeans who purpose coming to this country to complete their dental studies after a course more or less complete abroad, to report upon the value of such certificates, and how much credit should be allowed them in American dental colleges as a consequence, and to communicate to the Chairman of the Foreign Relations Committee any further facts that may serve as a guide to the deans of American dental colleges in the reception and proper assignment of such students.

*Fourth.* To furnish the Foreign Relations Committee with the names of such persons as may have come, or who may purpose coming, to the United States for professional instruction, and whom they may believe to be unworthy reception in American colleges, with the facts upon which such belief is based.

*Fifth.* To obtain for the Foreign Relations Committee, as far as is practicable, a complete list of all American graduates practising in Europe, giving names of the schools that issued their diplomas, together with date of graduation and the general reputation and status of such graduates.

The Foreign Relations Committee desires explicitly to say that while it is not authorized to extend the scope of its present action, and deems it unwise on its part to go further in defining the duties of the European Advisory Board, it is heartily in sympathy with American dental graduates abroad in their efforts to obtain a due recognition of the American dental degree in Europe, and pledges itself, whenever it believes the time is ripe for definite action, to take any steps which in its opinion will tend to bring about so desirable an object.

The Committee desires to announce the following appointments to the European Advisory Board:

Great Britain, Dr. W. Mitchell, London; Holland and Belgium, Dr. J. E. Grevers, Amsterdam; Denmark, Norway, and Sweden, Dr. Elof Förberg, Stockholm; Russia, ——— ; Germany, ——— ; Austria and Hungary, ——— ; Italy and Greece, Dr. Albert T. Webb, Rome; France, Dr. J. H. Spaulding, Paris; Spain and Portugal, ——— ; Switzerland and Turkey, Dr. L. C. Bryan, Basle.

S. H. Guilford,
J. D. Patterson,
T. W. Brophy,
H. W. Morgan,
W. C. Barrett, *Chairman,*
*Committee.*

208 Franklin Street, Buffalo, N. Y., U. S. A.

Copy of letter sent by Dr. Barrett to the President of the Chicago, Northwestern, and Lake Forest Universities after their action upon the matters made public in the Report of the Foreign Relations Committee of The National Association of Dental Faculties at Omaha, August, 1898:

Buffalo, N. Y., January 10, 1899.

President —— —— :

Dear Sir,—Some time since the Dental Colleges of the United States organized what is known as "The National Association of Dental Faculties," for the promotion of the best interests of dental education, for raising by combined action the standard of the requirements in preliminary education for matriculation in dental schools, and for the object of securing some uniformity of purpose in the conferring of the distinctively American degree of "Doctor of Dental Surgery."

Two years ago a standing committee was appointed, to be called the "Committee of Foreign Relations," of which I have the honor to be the chairman. Its functions are to take into consideration the interests of the large number of American college graduates practising in Europe, to endeavor to put a stop to the conferring of the American degree upon unqualified foreigners, and to take any practicable steps towards the suppression of the fraudulent colleges which sell dental diplomas for a mere money consideration and without attendance.

It is a fact that the nefarious business of vending dental degrees has been carried on to so great an extent that in nearly or quite all of the European countries enactments have been passed absolutely forbidding the use of the American title of "Doctor of Dental Surgery," and making it a penal offence for any one to attempt dental practice under such qualification. Formerly foreigners flocked to our dental colleges for the purpose of securing training in American dental methods, which were admittedly superior to those of most other countries. This has almost entirely ceased, because the American degree has been made a badge of disrepute in the minds of many foreigners through the infamous vending of diplomas by fraudulent dental colleges in this country.

Through her vicious legislation the State of Illinois has become the seat of this national scandal. I need not recall to your mind what specific law is referred to. Your recent action shows that you know it but too well, and that you are fully aware of the necessity for some action in vindication of the good name of the State.

As a teacher in an Illinois Dental College, I fully appreciate the opprobrium that rests upon the State while such a condition exists, and I write this to say that the Committee of which I am chairman has commenced legal proceedings to bring to justice some of those swindlers. As nearly as we can determine, there are at least a dozen pretended colleges in the city of Chicago which irregularly confer our dental degree. There are as many more charters in existence, conferring upon irresponsible men the power legally to grant degrees of all kinds, and they may begin the vending of diplomas to-morrow. The National Association of Dental Faculties stands ready in any practicable manner to co-operate with any body of men who will work towards obtaining the repeal of the Illinois laws which legalize this business.

Surely there is no body of men who could wield so powerful an influence in this good work as the presidents of famous Illinois universities and colleges, and there is none which has so vital an interest in maintaining the good name of the educational institutions of the State of Illinois. Will you not, then, as a member of that distinguished body of educators, allow us to proffer any assistance which the dental colleges of America can render. Our dental degree has been more extensively the sufferer from existing conditions than any other, because it is comparatively new, and because the institutions which properly confer it are in many cases independent of university affiliations, and the counterfeiting of their honors may therefore be carried on with a greater degree of impunity.

It seems to us that the first step to be taken should be the obtaining of the repeal of the mischievous Illinois law which permits fraudulent colleges to be chartered, and allows them to attach to their circulars and advertisements, with which foreign countries are flooded, the certificate of the Secretary of State of Illinois that an institution that openly offers for sale degrees in dentistry and other science is legally incorporated, and before the law is the peer and equal of the Chicago, Northwestern, and Lake Forest Universities. In the accomplishment of this good work I can tender to you the best efforts of the National Association of Dental Faculties, and the influence of the whole dental profession of the United States. I hope that it may be possible for us in some manner to be of use, and that the labors of different educational bodies, and of the teachers in all the fields of literature and science may be so concentrated as to insure success.

I am very truly yours,

W. C. Barrett,

*Chairman Committee National Association of Dental Faculties.*

*Answer of President Rogers to Dr. Barrett's letter.*

Northwestern University,

Evanston, Ill., January 14, 1899.

W. C. Barrett, M.D., Buffalo, N. Y.:

Dear Sir,—I beg to acknowledge the receipt of your very kind letter of the 10th inst. I have read it with a great deal of interest. I beg to assure you that we shall leave no stone unturned in this State to secure from the Legislature suitable legislation for the conservation of our educational institutions and the repute of our degrees.

I incorporated into my address at Springfield a portion of the report made by your Committee on Foreign Relations of the National Association of Dental Faculties, and also extracts from your letter. I intend to print the address, and shall take great pleasure in forwarding a copy of it to you. I shall also secure from the most prominent men of the State letters endorsing the movement which we have instituted, and shall print them in pamphlet form and put them into the hands of the Legislature. I shall be very glad to make use of a portion of your last letter to me in that communication.

Again thanking you, I am,

Yours very truly,

HENRY WADE ROGERS.

*Answer of President McClure.*

LAKE FOREST UNIVERSITY,

LAKE FOREST, ILL., January —, 1899.

DEAN W. C. BARRETT, M.D., Buffalo, N. Y.:

MY DEAR BARRETT,—It is a great pleasure to receive your letter of January 21. Dr. Rogers, of Northwestern, wrote me upon the receipt of my letter, in which I had enclosed your letter to myself, stating that he had already heard from you.

I am very willing to have you use the letter which I write you in any way that you deem wise. I am so heartily interested in all this work that I am eager to do everything within my power to accomplish the ends that we all seek. Allow me to thank you for having written me so fully with reference to your desire to bring about a high standard of preliminary education as a requirement for matriculation and graduation in our dental colleges. I am heartily in sympathy with you in this purpose. Students who have graduated at our Lake Forest College, and then have entered professional schools, always report to me that their preparation for their professional study is so much better than that of those who have not had these earlier privileges that they not only see their own superiority, but they also recognize the fact that their progress is retarded by the presence of those who are insufficiently prepared. Just so soon as we can increase the requirements for entering the dental college, just so soon we shall exalt the nobility and value of the dental profession as a profession. It seems to me that education on this subject will, in a brief time, bring about the results that are sought. With great respect and with great appreciation, I am,

Sincerely,

JAMES G. K. MCCLURE.

The following has been sent out by the State Board of Health to Medical Societies for their adoption:

January 2, 1899.

THE HONORABLE JOHN R. TANNER, *Governor of Illinois:*

DEAR SIR,—For the sake of the fair name of the State of Illinois, if for no other reason, it is imperative that the Act of 1872, concerning corporations, be amended, so that charters for educational institutions can no longer be issued under the provisions of this Act.

For the past twenty-six years it has been possible for any three or more persons to obtain a charter from the Secretary of State, for a nominal fee, for the organization of any institution for an educational purpose. As a result of this, there are at present in the State over two dozen fraudulent educational institutions, aptly termed "diploma mills,' which confer degrees in medicine, pharmacy, law, dentistry, divinity, arts and sciences, etc., upon any applicant in Illinois, in the United States, or anywhere in the inhabitable world, who possesses the necessary fee. This varies from five dollars to one hundred dollars, depending entirely upon the credulity and gullibility of the applicant.

Against this disgraceful and deplorable state of affairs the Illinois State Board of Health has for several years protested in vain. The law gave the Secretary of State no discretion in the issuance of charters, it being obligatory upon him to grant such when an application was properly made and the necessary fee paid. In 1885 the Board adopted a resolution asking that the Act concerning corporations be amended so as to exclude the practice of medicine from among those pursuits or objects for which corporations may be formed. This resolution was forwarded to the Legislature then in session, but without result.

These fraudulent concerns, every one of which is, to the utmost disgrace of the State, a legally chartered institution, have issued many hundred diplomas conferring the degree of Doctor of Medicine, and, as under the laws of many States and Territories of the

Union, graduates of legally chartered medical colleges could practise without restriction, and the holders of these diplomas become at once legally qualified practitioners. As an illustration, I will state that any ignorant or unscrupulous person in the State of Illinois can to-morrow, if he has ten dollars or less, obtain a degree of Doctor of Medicine in Chicago. With this degree he can, the day following, commence the practice of medicine in Detroit, Mich., and be entitled to all the rights and privileges which are accorded to the holder of a degree from any university in the United States or Europe.

As can be expected, this traffic has disgraced the State of Illinois at home and abroad, and has injured the standing of its educational institutions throughout the world.

As an example of the opinion entertained in the United States concerning this matter, let me quote the following:

"The State of Illinois is a glaring example of this kind of vicious legislation, and nearly or quite all the fraudulent colleges are now located in the city of Chicago.

"That city contains some of the best of our professional educational institutions, and at the same time the most villanous impostures conceivable.

"The citizens of other States are powerless, for Illinois is supreme within her own jurisdiction, and she continues to protect her criminals in their villany. The task of securing the repeal of the vicious law is too great for the courage of its reputable men, for ignorance and vice have struck hands in its maintenance." (From Report of the Committee on Foreign Relations of Dental Faculties, Omaha, August, 1898.)

"Illinois has disgraced the United States more than any State in the Union." (Henry Wade Rogers.)

"This . . . should make Illinois hang its head in shame, for so long as the Legislature permits such fraudulent institutions to flourish, the stigma is on the State." (Chicago *Times-Herald.*)

As has been shown in the Reports of this Board, relief can easily be obtained if legislation were enacted placing the issuing of charters of educational institutions with some educational body in the State, which will have the power also to amend or revoke the charters for cause at their discretion. This power to revoke those already issued is absolutely essential, for there are very many charters in existence which can be made available for many years to come.

This Board has suggested that the Board of Trustees of the State University would probably be the most suitable board in existence to carry out the provisions of such legislation. The Illinois Teachers' Association has adopted a resolution asking for the establishment of an Educational Commission, which will be vested with the right to grant and to deprive institutions of the degree-conferring rights.

Trusting that you will lend your influence to abolish this evil, I am,

Very respectfully,

—— ——.

Extract from report presented to the North Central Association of Colleges and Preparatory Schools, April 1, 1898, by President Henry Wade Rogers, of Northwestern University:

The committee submits the following recommendations:

1. That in each State represented in the Association an effort be made at the earliest opportunity to establish by law a body to be known as "The Educational Commission of ———" (inserting the name of the State).

2. That the Commission be composed of not less than six members, nor more than nine.

3. That the members of the Commission be appointed by the governor and confirmed by the senate. That no person be eligible to appointment on the Commission who is a member of the faculty, or board of trustees, or other governing body, of any educational institution within the State. And that membership in the Commission be forfeited *ipso facto* if at any time subsequent to the appointment aforesaid the person so appointed becomes connected with any educational institution in the manner above mentioned.

4. That the members of the Commission hold office for a period of not less than six years. And that the term of office be so arranged that not more than one-third shall retire in any one year.

5. That institutions hereafter incorporated shall derive the degree-conferring power from the Commission, and not otherwise. That institutions heretofore incorporated, and which now possess the degree-conferring power, may continue to exercise the same unless deprived of the right so to do by the Commission on the ground that the Institution affected falls below the standard which the Commission has established.

6. That the Commission shall not grant the degree-conferring power to any institution incorporated as a business enterprise, or to any one in which any part of the assets or income can be divided among stockholders, or to any institution having lower requirements for admission or graduation than the minimum standard therefore established by the Commission, or to any institution hereafter established as a college or university, unless its productive endowment shall amount to at least one hundred thousand dollars.

7. The Commission shall not confer the degree-conferring power upon any institution until such institution has applied therefor in writing, and accompanied the application with the sworn statement of the president and treasurer as to the amount of its productive endowment, the provision made for buildings, furniture, apparatus, and the requirements for admission and graduation.

8. The Commission shall have the right, after having given reasonable notice, to withdraw the degree-conferring power from any institution upon which it has conferred it, whenever an institution fails to meet the conditions necessary to justify the granting of the power in the first instance.

9. The Commission may require any institution to which it has granted the degree-conferring power to report under oath to it, at such times as it may designate, upon such matters as it deems necessary, to enable it to exercise intelligently the powers reposed in it. And the failure of an institution to report within a reasonable time and in a satisfactory manner shall justify the Commission in withdrawing from an institution, so offending, its degree-conferring power.

10. Any institution which exercises the degree-conferring power contrary to the provisions hereinbefore set forth shall forfeit its right to exist as an educational institution, and it shall be the duty of the law officers of the State to wind up its affairs. And the members of a board of trustees so offending shall be individually liable to fine, or imprisonment, or both, according to the discretion of the court.

Henry Wade Rogers, Northwestern University.
F. H. Snow, University of Kansas.
R. H. Jesse, University of the State of Missouri.
Joseph Swain, Indiana University.
George E. MacLean, University of Nebraska.
A. S. Draper, University of Illinois.
William F. Slocum, Colorado College.
George A. Gates, Iowa College.

